# Health Impacts of Per- and Polyfluoroalkyl Substances (PFASs): A Comprehensive Review

**DOI:** 10.3390/life15040573

**Published:** 2025-04-01

**Authors:** Csilla Mišľanová, Martina Valachovičová

**Affiliations:** Institute of Nutrition, Faculty of Nursing and Professional Health Studies, Slovak Medical University, 833 03 Bratislava, Slovakia; martina.valachovicova@szu.sk

**Keywords:** persistent organic pollutants, exposure, biological matrices, LC-MS/MS, health effects

## Abstract

Per- and polyfluoroalkyl substances (PFASs) are among the persistent organic pollutants characterized by their persistence in the environment, high mobility, and adverse impact not only on the ecosystem but also on human health. The biggest challenges in human biomonitoring are the low concentrations of PFASs in biological matrices and the presence of matrix interferents in samples. The combination of liquid chromatography with tandem mass spectrometry (LC-MS/MS) and solid-phase extraction (SPE) as a sample preparation technique appears to be the most suitable solution for achieving the desired selectivity and sensitivity in PFAS determination. The aim of this review is to describe possible sources of PFASs, their presence in various human matrices, analytical methods for determining PFASs in different biological matrices using various pretreatment techniques for complex samples, as well as adverse health risks associated with PFAS exposure. The most studied PFASs include PFOA and PFOS, which are most frequently detected in matrices such as plasma, serum, and breast milk. The average concentrations of PFOA range from 1.0 to 2.6 ng.mL^−1^ in plasma, 1.9 to 2.4 ng.mL^−1^ in serum, and 0.4 to 3.1 ng.mL^−1^ in breast milk. For PFOS, the average concentrations were 2.0–4.0 ng.mL^−1^, 3.7–4.6 ng.mL^−1^, and 3.6–4.8 ng.mL^−1^ for plasma, serum, and breast milk, respectively. The most significant health effects associated with exposure to long-chain PFASs (such as PFOA and PFOS) include lipid disorders, hypertension, diabetes mellitus, thyroid disorders, infertility, cancer, obesity, autism, neurodevelopmental issues, cardiovascular diseases, and kidney and liver disorders. It is of utmost importance to monitor PFAS exposure, predict their toxicity, and develop effective strategies to mitigate their potential effects on human health.

## 1. Introduction

Pollution caused by organic contaminants is becoming a global problem, affecting not only the environment but also human health. These contaminants include pharmaceuticals, pesticides, and poly- and perfluorinated substances (PFASs). PFASs are anthropogenic microcontaminants that, due to their widespread occurrence, have an adverse impact on the ecosystem and human health. The most studied compounds are perfluorooctanoic acid (PFOA) and perfluorooctane sulfonate (PFOS) [1,2,3]. Due to its potential multi-organ toxicity and harmful effects on human health, PFOS was the first compound to be regulated by the European Commission in 2006 (EU, 2006) [4]. It was listed in the Stockholm Convention on Persistent Organic Pollutants (POPs) in 2009 [5]. PFOA was listed in 2019 [6] and perfluorohexanesulfonic acid (PFHxS) in 2022 [7].

The first classification of PFASs was introduced by Buck et al. [8], who defined them as fluorinated aliphatic substances containing one or more carbon atoms, where the aliphatic hydrogens may be partially or fully replaced by fluorine atoms. In 2018, the Organisation for Economic Co-operation and Development (OECD) defined PFASs as substances that contain -CnF2n+1 or -CnF2n- (n ≥ 1). The existing classification system was further modified by Buck et al. [9] so that PFASs are divided into two groups: polymers and non-polymers. Non-polymers are divided into per- and polyfluorinated substances, with the perfluorinated ones having polar hydrophilic groups such as carboxylate (COO^−^), sulfonate (SO_3_^−^), or phosphate (OPO_3_^−^) mostly attached to a hydrophobic carbon chain. Polymers are divided into fluoropolymers, fluorinated side-chain polymers, and perfluoropolyether [10]. These polymers are not bioavailable and do not accumulate in the environment or biological systems. They gradually break down into PFASs.

PFASs do not degrade under normal environmental conditions but can undergo physicochemical changes to the formation of smaller alkyl chain compounds [11]. Long-chain PFASs are being replaced by new short-chain alternatives, such as perfluorobutanoic acid (PFBA), perfluorobutanesulfonic acid (PFBS), GenX (hexafluoropropylene oxide dimer acid [HFPO-DA]), ADONA (dodecafluoro-3H-4,8-dioxanonanoate), and F-53B (6:2 chlorinated polyfluoroalkyl ether sulfonate [6:2 Cl-PFAES] and 8:2 Cl-PFAES). Manufacturers claim that the replacement PFASs are not associated with adverse health effects. However, studies have shown that some of these substitutes may exhibit even higher toxicity [12,13].

Although alternatives to PFAS compounds have been in use for several decades, scientific studies on their concentrations in surface water remain limited [14].

GenX (a replacement for PFOA) is a perfluoropolyethercarboxylic acid (PFECA), which is used to produce perfluoropolymers as a polymerization aid. It is chemically stable, environmentally resistant, and has higher hepatotoxicity than PFOA, even at low concentrations, raising significant health concerns [11].

6:2 chlorinated perfluoroalkyl ether sulfonic acid (6:2 Cl-PFESA) and (8:2 Cl-PFESA), known as F-53B, have become alternatives to PFOS. Toxicological studies indicate that F-53B may cause endocrine disruption of the thyroid gland even at low environmental concentrations and has a higher bioaccumulation potential than PFOS. ADONA, a replacement for PFOA, has been applied as an emulsifier in fluoropolymer production [15].

Since both GenX and F-53B are major perfluorinated substances that pollute, they may pose an even greater risk than PFOA and PFOS. It is, therefore, essential to monitor and evaluate their potential toxicity in humans [16].

Currently, there is limited information on their reproductive toxicity and other long-term health effects. Further toxicological studies are needed to fully understand their impact on both the environment and human health.

Several studies have shown that alternatives to PFOA and PFOS are found in the environment at comparable or higher concentrations than PFOA and PFOS [16,17]. For example, concentrations of GenX in surface water samples are three times higher than PFOA in the North Sea [18]. Concentrations of F-53B in aquatic and soil environments in China are similar to those of PFOS [14,19].

The hydrophobic and oleophobic parts of PFAS compounds cause them to act as surfactants. They are widely used in various industries and consumer products, such as carpets, textile impregnations, fire-fighting foams, electroplating, adhesives, protective coatings, insecticides, household cleaners, cosmetics, electronics, explosives, food packaging, tea bags, and many others from the 1940s to the present. The United States Environmental Protection Agency (U.S. EPA) has set maximum concentrations of PFASs in drinking water at 4 ng.L^−1^ for PFOA and PFOS and 10 ng.L^−1^ for PFHxS in 2024. PFASs are ubiquitous substances found in various environmental compartments, such as air, surface water, drinking water, groundwater, sediment, soil, plants, food, and animals. Routes of exposure to PFASs by inhalation and dermal contact include dietary intake, indoor air, dust, and drinking water [20,21]. As a result, PFASs are found in multiple human tissues, such as blood, urine, hair, nails, urine, placenta, and breast milk.

This review offers insight into PFAS exposure pathways, their different behavior, and an overview of detection methods for analysis in biological matrices. At the same time, the review provides a summary of the potential harmful effects on human health.

## 2. Properties of PFASs

PFASs contain a hydrophobic chain of varying lengths made up of carbon and hydrogen atoms and a hydrophilic functional group at the end of the chain. The hydrogens can be fully or partially replaced by fluorine atoms. Accordingly, we divide the compounds into perfluorinated, when all hydrogen atoms are replaced by fluorine atoms, and polyfluorinated, when some hydrogen atoms (attached to at least one carbon) are replaced by fluorine atoms. According to the chemical structure, we divide PFASs into a long-chain group (number of carbons 7–13) called legacy PFASs and a short-chain group (number of carbons six or fewer atoms) term emerging or alternative compounds. Polyfluoroalkyl substances can be chemically modified into perfluoroalkyl substances [22].

**Figure 1:**
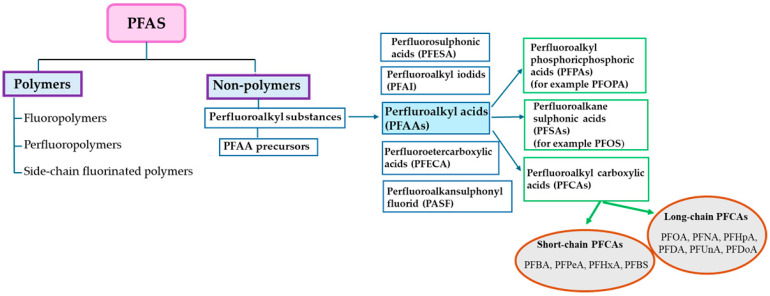
Classification of PFASs.

The most studied are the perfluoroalkyl acids (PFAAs), which include long-chain compounds, such as 9-carbon perfluorononanoic acid (PFNA), 8-carbon compounds PFOA and PFOS, 7-carbon perfluoroheptanoic acid (PFHpA), and PFHxS. PFAAs also include short-chain 6-carbon compound perfluorohexanoaic acid (PFHxA), 5-carbon perfluoropentanoic acid (PFPeA), 4-carbon perfluorobutanoic acid (PFBA), and perfluorobutanesulfonic acid (PFBS). The classification of PFASs is presented in Figure 1.

**Figure 2:**
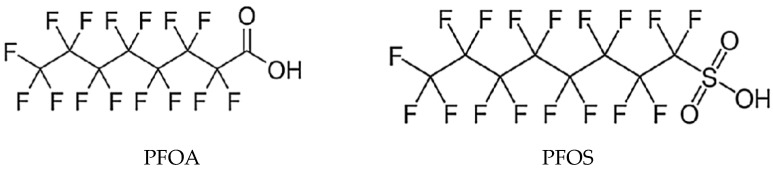
Molecular structures of PFOA and PFOS.

The most commonly monitored PFASs are PFOS and PFOA, whose molecular structures are shown in Figure 2.

PFASs consist of two parts, namely, an alkyl chain and a functional group, which can be a carboxylate or sulfonate. The alkyl chain, in which hydrogen atoms are replaced by fluorine, is responsible for the physical and chemical properties of PFASs. Fluorine has high electronegativity, ionization potential, and low polarization. It is the most electronegative element in the periodic table; therefore, the bond between carbon and fluorine is among the strongest covalent bonds with a dissociation energy of up to 513.5 kJ.mol^−1^. The strength of the bond increases with the number of F atoms bound to carbon atoms. The strong bond between C and F is responsible for the thermal stability of PFASs and their resistance to degradation, hydrolysis, and photolysis, which is related to their long-term persistence in various components of the environment but also in the food chain and in the human body [23,24]. They are further divided into polymers and non-polymers, with the polymeric ones degrading to non-polymers over time. Due to the low polarizability of fluorine atoms, PFASs exhibit hydrophobic properties, weak intra- and intermolecular interactions, and, as a result, higher volatility and lower boiling point. Long-chain PFASs have higher hydrophobicity, and stronger hydrophobic interactions occur compared to short-chain PFASs [25].

Because PFASs have different chain lengths and functional groups, they exhibit different physicochemical properties. Short-chain PFASs have higher leaching potential and higher mobility. Long-chain PFASs have higher hydrophobicity and lower mobility. The sulfonate and carboxyl functional groups in PFASs are responsible for their adsorption capacity, mobility, and solubility. The length of the carbon chain can affect the bioavailability and molecular interaction potential of PFASs, thus influencing long-term toxicity. They have low surface tension and thus good surface wettability. The oleophobic character is a consequence of weak van der Waals forces. The combination of hydrophobic and hydrophilic properties allows PFASs to interact in the environment. They have the ability to combine into micelles, and they are resistant to degradation and oxidation. Moreover, they persist in the environment for long periods and accumulate in various human organs. They are capable of long-distance transport through air, water, and the atmosphere [26].

Since PFASs have different chain lengths and different structures (such as branched or linear isomers) and functional groups, they exhibit different physicochemical properties that affect PFAS concentrations and their chemical behavior (bioaccumulation, transport) in surface water. Short-chain PFASs have higher leaching potential as well as higher mobility, while long-chain PFASs have higher hydrophobicity and lower mobility. Sulfonate and carboxyl functional groups in PFASs are responsible for their adsorption capacity, mobility, and solubility. Long-chain sulfonate groups show greater bioaccumulation compared to carboxylate groups and PFASs containing shorter carbon chains. The length of the carbon chain can affect the bioavailability and molecular interaction potential of PFASs and thus affect long-term toxicity. The most abundant short-chain PFASs in environmental samples are PFBS and PFBA. They are characterized by lower bioaccumulation potential, lower degradability, and greater mobility and persistence compared to long-chain PFASs [27].

The hydrophilic functional group of PFASs is responsible for their thermal and chemical stability, adsorption capacity, relatively high solubility, and mobility in aqueous media. The solubility of PFASs is influenced by the number of carbon atoms in the fluorinated compound—specifically, solubility decreases as the number of C–F groups increases.

The poor polarizability of fluorine atoms contributes to the dual hydrophobic and lipophilic nature of PFASs. As the chain length increases and hydrogen atoms are replaced by fluorine atoms, PFASs lose some of their chemical inertness. Short-chain PFAS compounds are more soluble in water, making their removal more challenging [25,26,27,28]. For long-chain PFASs, retention is primarily governed by hydrophobic interactions, whereas for short-chain PFASs, electrostatic interactions play a more significant role. Short-chain PFASs exhibit lower sensitivity to solution pH, limited bioaccumulation, rapid excretion, and selective elimination through electrostatic interactions. At low pH, they degrade more rapidly, leading to higher concentrations in groundwater compared to long-chain PFASs, which are more resistant to degradation. The degradation time of PFASs increases with carbon chain length. Higher temperatures promote microbial activity and biodegradation, as well as interactions with other contaminants, ultimately leading to a decrease in PFAS concentrations in surface water [27,29].

Based on functional groups, carbon chain length, and polarity, the mobility of PFASs in the solid–water system can be predicted. Negatively charged PFAS compounds exhibit higher mobility than neutral or positively charged PFASs, which reduces the likelihood of groundwater contamination. Long-chain PFASs tend to partition into more hydrophobic phases in water, such as the organic carbon component of suspended particles, but they also readily bind to positively charged ions associated with dissolved organic carbon. Sorption of PFASs at the sediment–water interface reduces their mobility in groundwater. The solubility of PFASs in water and their polarity are not significantly influenced by the strong polarity of the C-F bonds [30].

Their high mobility provides the possibility of being transported over long distances, for example, to remote areas such as the Arctic or Antarctica. PFASs are soluble in both non-polar and polar solvents, and their boiling and melting points increase with chain length. PFASs with higher vapor pressure exist in the gaseous state, and those with lower vapor pressure remain solid or liquid [23,24].

The most commonly monitored physicochemical parameters include pH, temperature, turbidity, salinity, electrical conductivity (EC), dissolved oxygen (DO), and environmental factors (such as chain length, seasonal variations, organic content, solubility, partition coefficient, redox potential) that affect the fate, mobility, and occurrence of PFASs in the aquatic ecosystem. Available data indicate that high pH, turbidity, solubility, and short-chain carbon lengths can lead to higher concentrations of PFASs in water. Conversely, high electrical conductivity, temperature, salinity, and sediment/water partition coefficients can result in lower concentrations of PFASs in water. Future studies should focus on the long-term monitoring of PFASs in water to assess the effects of these parameters on environmental conditions, as well as the influence of physicochemical properties on potentially emerging contaminants [23,24].

The study by Ohoro et al. found that at high pH, turbidity, and dissolved oxygen, PFOS concentrations are high. Conversely, high electrical conductivity, temperature, and salinity cause a decrease in PFOS concentrations in water. The protection of aquatic animals with regard to environmental safety, as well as human safety, is necessary [31].

## 3. Sources of PFAS Exposure

Due to the high abundance of PFASs in the environment, there are multiple routes of human exposure to PFASs, with the main sources of exposure being drinking water, air, indoor dust, soil, food, food contact materials, cooking utensils, mother-to-fetal transmission, and breastfeeding of newborns. However, it is expected that 98% of the human population in the U.S. may have detectable levels of PFASs in their blood. Given the potential risks to human health from PFASs, the United States Environmental Protection Agency (U.S. EPA) and the International Agency for Research on Cancer have classified certain PFASs as probable human carcinogens. These substances are also associated with endocrine disruption, genotoxicity, immunotoxicity, reproductive toxicity, and hepatic toxicity [32].

In the adult population, food and drinking water are the main sources of exposure, but this is influenced by overall lifestyle and the level of contamination of drinking water. Infants fed formula may be among the most exposed due to high water intake per body weight [32,33,34].

Due to their exceptional properties, such as stability, water resistance, etc., they are widely used in industry and commercial products. They can enter the environment through various routes: industry, emissions, product use, and lividation, and the result is the insufficiently effective removal of PFASs by wastewater treatment plants, resulting in soil and water contamination. Their degradation and removal from the environment depend on the properties of PFASs, especially the chain length, type of head group, degree of ionization, and fluorination.

Accumulation in humans occurs through dermal contact, inhalation, or ingestion and can cause organ-specific toxicity, such as hepatotoxicity, neurotoxicity, immunotoxicity, etc. Long-chain PFASs accumulate in the body for a longer period of time and can result in health problems such as hormonal imbalance, decreased immune function, liver damage, various developmental and reproductive anomalies, and an increased risk of cancer. The toxicokinetic profile of PFASs in humans consists of absorption, distribution, metabolism, and excretion [35]. PFAS absorption can occur orally, inhaled, and dermally. The oral route predominates (>90%), mainly through contaminated food and drinking water [36]. Inhalation can occur when handling consumer products or in work environments where products containing PFASs are manufactured [37]. Dermal contact occurs when using personal care products or when coming into contact with surfaces contaminated with PFASs [38]. The rate and extent of absorption in the human body depend on the physicochemical properties of PFASs, namely molecular weight, chain length, functional groups, hydrophobicity, lipophilicity, solubility, partition coefficient, and volatility. Short-chain PFASs, such as PFBS and PFHxA, are more soluble in water than long-chain PFOS and PFOA, which bioaccumulate in the body over a longer period of time [39].

In addition to food and water intake, inhalation of indoor contaminated air and dust are also important routes of exposure to PFASs through ingestion or skin contact. Dust can act as a reservoir for many chemicals used in various consumer products. Quantifying PFASs in indoor dust is essential, as higher concentrations of PFASs have been measured indoors compared to outdoors [40].

Most data on adverse health risks are related to long-chain PFASs. However, there are growing concerns about the potential adverse effects of short- and ultra-short-chain PFASs. This is particularly important to consider for pregnant women, infants, and children, who are the most vulnerable groups and particularly sensitive to PFAS exposure due to their higher absorption rates [41].

Research investigating the correlation between PFAS exposure and fetal sex hormone levels indicates that male and female infants exhibit differences in the function and concentrations of sex hormones in their serum. Therefore, it is important to consider the sex-specific nature of the relationship between PFAS exposure and hormone levels [42].

In a study by Høyer et al. [43], the association between prenatal exposure to PFHxS, PFHpA, PFNA, PFDA, PFOA, and PFOS and behavior and hyperactivity in 5–9-year-old children from Greenland and Ukraine was investigated. A country-specific parental standardized questionnaire was used to assess behavior in 1023 children. Parents rated their children’s behavior over the past six months. PFOS exposure levels were higher in Greenland than in Ukraine. Foods such as fish, seabirds, and marine mammals, which have high levels of trace elements, are a major part of the diet. This fact explains the differences in PFAS exposure. The results suggest that some of the associations between PFOS exposure and hyperactivity in some children may be due to postnatal exposure through breastfeeding. In the case of learning disabilities in children, a more detailed investigation will be needed as levels of PFHxS and PFNA increase with decreasing PFOS levels [44].

In addition to legacy PFOA and PFOS, which have been widely detected among pregnant women in Asia, North America, and Europe, some short-chain PFASs, such as PFBS, with comparatively shorter half-lives, are emerging. However, these substances may still have adverse effects on human health. The shift from long-chain to short-chain PFAS production has caused changes in the relative distribution of PFASs in serum, but it remains unclear whether chronic exposure has had a negative impact on pregnancy [43].

Bharal et al. reviewed the persistence of PFASs, exposure routes, and toxicological profiles, providing a comprehensive analysis of neurotoxicity studies and their mechanisms induced by PFAS exposure [45].

### Removal of PFASs

Separation and concentration techniques are very important in the remediation of PFAS-contaminated waters because these techniques will result in an increase in concentration and a decrease in the volume of liquid for which a destruction process can be used. Knowledge of the physicochemical properties of PFASs is essential for remediation technologies. Removal of long-chain PFASs from contaminated water can be achieved by non-destructive (using granular activated carbon, ion exchange resins, and reverse osmosis), destructive (biodegradation, incineration, advanced oxidation, etc.), and hybrid technologies (combining two or more treatment processes). Membrane filtration has an efficiency of >99%, but its disadvantage is energy consumption [46].

Therefore, future research should focus on low-cost, energy-efficient technologies for the removal of short-chain PFASs. Furthermore, research into safer alternatives to PFASs could contribute to the phasing out of PFASs. Since PFASs tend to accumulate in the food chain and in animal tissue, their degradation is either slow or impossible under normal conditions. Short-chain perfluoroalkyl acids (PFAAs) such as PFPeA, PFHxA, and PFHpA are less persistent in humans, but their potential toxicity, environmental persistence, and exposure impacts are still not well studied. Future research should focus on the use of sustainable technologies for their removal [47].

## 4. Analytical Techniques

The chemical structure of PFASs allows them to interact with the environment, which can result in toxic effects on humans and the environment, and therefore, monitoring the impact of PFASs on human health is essential. Biomonitoring of PFASs helps in elucidating the pathway by which these chemicals negatively affect humans and in assessing risks to human health. The main challenges of PFAS analysis in biological samples include complex matrix interference, low target concentration, the existence of multiple PFAS homologs and isomers, and poor sample stability. Due to the relatively low concentration of PFASs in biological matrices and the complexity of the sample, continuous improvement of comprehensive and highly sensitive analytical methods is necessary, especially in increasing the sensitivity and robustness of the method.

A review article by Kee et al. [48] describes human sample collection, sample preparation techniques, and instrumental techniques for sample analysis. When collecting, storing, and analyzing samples, the use of sampling material (tubes, sample containers, sampling tools) made of Teflon^®^ (polytetrafluoroethylene, PTFE) should be avoided. Polypropylene (PP) and polyethylene (PE) materials are recommended. Since PFASs have the ability to adsorb to glass surfaces in aqueous environments, the use of glass devices should be avoided [49].

Chromatographic methods, including liquid chromatography (LC), high-performance liquid chromatography (HPLC), ultra-high-performance liquid chromatography (UPLC), gas chromatography (GC) combined with tandem mass spectrometry (MS/MS), are most commonly used for the analysis of PFASs [50].

LC-MS is one of the most widely used techniques suitable for the separation of both neutral and ionic PFASs in various human samples (blood, plasma, serum, urine, hair, nails). The most commonly used mass spectrometers are triple quadrupole (QqQ), which are sensitive, selective, and provide accurate detection and quantification of PFASs at low concentrations, especially for targeted analyses in complex matrices. Due to the ionic nature of many PFASs, electrospray ionization (ESI) in negative mode is most commonly employed as the ionization method [51].

However, during the quantification of PFASs in biological samples using HPLC-MS or MS/MS, matrix effects are often observed. These effects can lead to ion suppression or enhancement, loss of ionic intensity of the target analyte, or false signal increases or decreases, which can significantly affect the sensitivity and accuracy of the analytical method. Electrospray ionization (ESI) is more susceptible to matrix effects due to its ionization mechanism than atmospheric pressure chemical ionization (APCI). To eliminate matrix effects, it is necessary to use isotopically labeled standards or matrix-matched calibration [52,53].

High-resolution mass spectrometry (HRMS) is employed for non-targeted analysis to identify unknown PFASs at trace levels. Mass spectrometers (e.g., QTOF-MS, Orbitrap MS) are characterized by high selectivity, sensitivity, and resolution, allowing them to distinguish interferents present in complex matrices. Moreover, HRMS provides accurate mass and empirical formula information, which helps ensure reliable interpretations of unknown compounds. Compared to MS/MS, HRMS can avoid false positive results that may arise when only one product fragment or one multiple reaction monitoring (MRM) transition is used in the LC-MS/MS experiment [54,55,56].

The advantages of quadrupole, linear ion trap, and Orbitrap analyzers are combined in the hybrid Tribrid Orbitrap, which can identify and quantify unknown PFASs [57].

Matrix-assisted laser desorption/ionization (MALDI) mass spectrometry has also been used to quantify PFASs in complex biological matrices, where efficient desorption and ionization are achieved using a stable, low-temperature plasma within a few seconds. In addition to traditional methods, ambient dielectric barrier discharge ionization (DBDI-MS) is used for the direct and sensitive detection of ionized volatiles in complex mixtures, with efficient desorption and ionization also achieved using a stable, low-temperature plasma within seconds.

Fluorescence detection exploits the interaction between fluorescent materials and analytes to detect target analytes. Fluorescence sensing methods use various fluorescent materials as signal probes [50].

Less frequently, gas chromatography (GC) combined with a flame ionization detector (FID), an electron capture detector (ECD), or a mass spectrometer (MS) has been used as well [58]. GC-MS is a suitable method for the determination of volatile, semi-volatile, and neutral substances but is not the preferred technique for the determination of PFASs due to their polarity and difficulty in evaporation. Therefore, a derivatization process is necessary before the GC analysis itself. Although GC is advantageous over LC for the identification of PFAS isomers due to the higher number of potential separation plates, the determination of PFASs in biological samples using GC is characterized by high LOD, long analysis time, and a lengthy derivatization process [59,60,61].

Eliminating matrix interferences and simultaneously increasing the detection limit of PFASs in various complex matrices are essential steps in the analysis of PFASs. Various extraction techniques are used for sample preparation. Review Dhiman et al. deals with different extraction techniques, analysis, and detection of PFASs in different matrices [62].

For an accurate assessment of PFAS exposure, proper matrix selection is necessary. Biomonitoring matrices can be invasive (blood, serum, breast milk, tissues) and non-invasive (hair, nails, skin, urine). Plasma and serum are most commonly used for practical reasons.

Since invasive matrices have a number of disadvantages, such as making it difficult to collect samples from infants, children, pregnant women, and the elderly, non-invasive matrices are preferred. Urine is suitable for monitoring short-chain PFASs. Highly volatile PFASs do not accumulate in hair, and PFAS levels in breast milk may not accurately reflect maternal exposure because breastfeeding may affect the rate of PFAS excretion [63].

The process of sample pretreatment prior to instrumental analysis is a purification method used to reduce instrumental contamination, signal interference, and matrix effects resulting from interfering substances in complex real-world samples. Pretreatment for PFAS analysis presents challenges for several reasons: (1) PFASs are often found in real-world samples at low concentrations and often coexist with other hydrophobic substances. (2) Extraction of PFASs from various sources, including food, environmental, and biological samples, becomes challenging in the presence of complex matrix interfering substances (e.g., proteins, lipids, polysaccharides, vitamins, and others).

Sample pretreatment includes techniques ranging from simple ones, such as filtration and dilution, to more complex ones, such as different extraction methods. The most commonly pretreatment techniques are solid-phase extraction (SPE) (using mainly hydrophilic–lipophilic-balanced (HLB) sorbents; weak anion exchanger (WAX); polymeric cartridges; polyacrylonitrile and divinylbenzene/carboxen/polydimethylsiloxane fibers) [64]; liquid–liquid extraction (LLE) [65,66]; solid–liquid extraction (SLE) [67]; ultrasound-assisted extraction [68,69]; dispersive solid-phase microextraction [70]; dispersive liquid–liquid microextraction [71]; or accelerated solvent extraction (ASE) [72]. SPE was the most commonly used pretreatment technique, and it was applied in about 75% of the studies [73].

A review article by Comito et al. [74] provides an overview of selected analytical methods for the determination of PFASs in various biological matrices as well as sample pretreatment in the period 2003–2022.

In this review, Table 1 presents selected examples of PFAS determination in conventional biological matrices after 2022.

Nuclear magnetic resonance (NMR) is not sensitive to matrix components and offers clean spectra. It allows PFASs to be quantified without requiring high reproducibility and cost-effectiveness [95,96]. Advanced and novel analytical methods include fluorimetric assays, which measure the fluorescence signal of common fluorophores in solution. An interesting technique is molecular imprinting, which combines fluorescent dyes with molecularly imprinted polymers (MIPs). Electrochemical techniques are also used to detect PFASs, which help increase the sensitivity of the method [97].

## 5. Human Matrices

Measuring chemicals or their metabolites in different matrices is important for assessing human exposure and associated risks. About 77% of all published articles concerned blood, plasma, and serum, as they best reflect internal exposure to PFASs. The disadvantage is the invasiveness of the collection, for example, in newborns and children, due to ethical and humanitarian reasons, which limits the number of available samples. Therefore, non-invasive matrices such as hair and nails have several advantages, including simple and easy sampling, transport, stable storage, and ethical acceptance. Moreover, hairs contain keratin, which is suitable for monitoring PFAS exposure. Another non-invasive sample is urine. Each matrix has its advantages and disadvantages. Hair and nails require simple collection, but due to external transfer, they may contain various impurities that need to be removed [98].

### 5.1. Hair

Hair as a non-invasive matrix and contains 15–35% water, 63–95% protein, and 1–9% lipid, making it a suitable reservoir for hydrophobic compounds and their hydrophilic metabolites [89,90,91,92,93,94,95,96,97,98,99,100]. The review article by Zhang et al. describes standardized procedures for measuring microorganic contaminants (MOCs) in hair, sample collection and preparation, methods for determining chlorinated persistent organic pollutants, brominated flame retardants, non-persistent pesticides, per- and polyfluoroalkyl substances, phthalate esters, bisphenols and polycyclic aromatic hydrocarbons in hair, and conclusions from cohort and epidemiological studies. There are several factors that reduce the reliability of hair analysis. Detection of MOCs is affected by age, gender, hair treatment, and sampling method. Furthermore, it is necessary to distinguish between endogenous and exogenous contamination in hair, either by incorporation via blood or from the environment. Organic pollutants can be incorporated into hair through three routes: internal passive diffusion via the bloodstream, external transfer to the hair structure from sweat and sebum, and absorption from the external environment [101,102].

Correlations between MOC concentrations in hair and in blood/urine are limited. Since hair has been less used in the past to assess the health risk of contamination with microorganic contaminants compared to blood/urine, there is a need to discuss techniques and experiments and propose standardized procedures for their measurement [82].

In a study by Wang et al. [103], concentrations of PFOS, PFOA, PFHxA, PFNA, PFDA, PFUnDA, PFDoA, and PFHxS were determined in 39 human serum, urine, hair, and nail samples by HPLC-MS/MS. PFHxA was detected only in nails, and PFDoA was found in urine, hair, and nails at very low concentrations. The concentrations of PFOS were 9.24 ng.mL^−1^ in serum, 13.96 ng.L^−1^ in urine, 0.58 ng.g^−1^ in hair, and 0.63 ng.g^−1^ in nails and were higher in all matrices compared to other PFASs. A statistically significant difference in serum concentrations of PFOA and PFHxS was found between men and women. However, no statistically significant difference in PFAS concentrations was found between older and younger individuals in all matrices. The results of the study showed that the most suitable matrix for monitoring PFASs, especially PFOS, was nails [103,104].

PFASs can enter the hair in three ways: internal passive diffusion through the bloodstream, from the external environment, and external transfer into the hair shaft from sweat and sebum [102]. The memory effect of hair due to the accumulation of chemicals allows for retrospective analysis. In the case of hair, it is possible to increase the frequency of sample collection, as well as its amount, which is especially advantageous for toddlers. In an Indian study [105], the concentrations of 25 PFASs (6 PFSA, 13 PFCA, and 6 PFAA precursors) were determined in 39 human hair samples. The effect of gender was also monitored. PFOA, PFOS, and PFHxS were the most abundant in the samples. A highly significant correlation was found between PFOS and PFHxS. Women showed a higher incidence of PFASs than men. The article summarizes the results of PFAS analyses in hair obtained around the world. Compared to global results, PFAS values in the Indian study reached lower concentrations [105].

The review article by Robin et al. presented the development and optimization of the method, including sample collection and preparation, extraction procedures, and instrumental techniques. Regarding hair sampling, the preferred site is the back of the head (posterior vertex, occipital region, or neck) or as close to the scalp as possible. Aluminum, envelopes, polypropylene tubes, or glass vials are used for hair storage [106].

Samples were mainly stored at room temperature, but storage in a refrigerator or freezer has also been reported. Combinations of aqueous and organic solvents, aqueous solvents, organic solvents, and shampoo were used for hair decontamination. Hair samples were dried at room temperature or lyophilized. After external decontamination of the sample, hair was cut into small pieces or ground into powder. The samples were denatured with an organic solvent, most often methanol, or by acid hydrolysis (HCl, acetic, formic, nitric acid) and denaturation followed by purification [101,107]. In general, LC-MS/MS or GC-MS/MS are applied for the determination of polar or non-polar compounds, respectively. Prior to instrumental analysis, matrix interferents have to be removed, usually by extraction methods (LLE, SPE, or SPME) [108,109,110].

A Spanish study was aimed to determine PFBuA, PFPA, PFHxA, PFHpA, PFOA, and PFOS in hair samples from children, women, and men, assessing the potential relationship between PFAS concentration and age, gender, smoking, and hair dyeing or coloring. The samples came from 42 volunteers, of whom 10 were children, 16 were women, and 16 were men. The concentrations of PFASs ranged from 0.6 to 15.5 ng.g^−1^, with PFHpA and PFOS being present in at least 86% and 76% of the analyzed samples, respectively. Longer-chain PFASs, such as PFOA and PFOS, are more commonly detected in hair samples, while shorter-chain PFASs (PFBuA) are more commonly detected in urine [111].

Hair and nail samples offer the advantage of easy sampling, but both can incorporate pollutants through external transfer. Therefore, they require an additional washing/rinsing step using a solution of surfactants in water or a hydrophilic organic solvent to determine internal exposure [102].

### 5.2. Placenta

The placenta is an important organ for the transfer of oxygen and nutrients between the mother and fetus. Various studies have focused on the capacity of the placenta to transfer PFASs and their alternatives. In a Chinese study, Pan et al. included 100 paired samples of human maternal and umbilical cord serum and found that chlorinated polyfluorinated ether sulfonates (6:2 and 8:2 Cl-PFESA) were detected in more than 99% of both matrices. Higher efficiency of placental transfer was associated with older maternal age, higher education, and lower glomerular filtration rate [111].

Gao et al. presented an analysis of 132 paired samples of maternal and umbilical cord serum. The study showed that the extent of placental transfer of short-chain PFASs was in the range of 97–146% [112].

A review article by Blake et al. [113] focused on the placenta as a target organ and adverse pregnancy outcomes due to PFAS exposure. Specifically, these include prolonged gestation, pregnancy-induced hypertension, preeclampsia, gestational diabetes, and low birth weight. Studies by Chen [114] and Wang et al. [115] in human and animal models have shown that PFASs readily pass from maternal serum through the embryo to the placenta, which in the early stages functions as the liver, lungs, and kidneys for the embryo. Wang analyzed PFHpA, PFOA, PFNA, PFDA, PFUA, PFDoA, PFBS, PFHxS, PFOS, and PFOSA in 369 pairs of maternal and cord serum in China. Almost all maternal and cord serum samples were found to contain all PFASs analyzed. All ten PFASs were found in both mothers and cord serum in almost all samples. Maternal and cord levels were closely correlated (r = 0.485–0.908) for all PFASs except PFBS. The efficiency of transplacental transfer (TTE) was influenced by carbon chain length and functional group. Perfluoroalkylsulfonates had a lower maternal–fetal transfer ratio compared to perfluoroalkylcarboxylates [115].

In a review article, Liu et al. summarized the findings from previously published data on PFAS monitoring in maternal blood, cord blood, breast milk, placenta, amniotic fluid, fetal organs, dried blood spots of newborns, and infant serum and on PFAS exposure during pregnancy and breastfeeding. PFAS concentrations determined in blood are relatively high, while in breast milk they are relatively low. They emphasized the importance of future research on PFAS isomers and enantiomers [116].

In a Chinese study, 50 pairs of maternal and umbilical cord serum samples as well as placenta obtained from pregnant women living around the fluorochemical industrial park in Fuxin were tested. A total of 49 target PFASs in 11 classes were identified in human samples by HPLC-MS/MS [100]. PFBS, PFBA, and PFOA were the main contaminants from 21 target analytes of legacy PFASs in maternal and cord serum samples, as well as placentas analyzed by HPLC-MS/MS. PFBS concentrations in maternal serum ranged from 3.6 to 140 ng.mL^−1^, PFOA from 0.08 to 123 ng.mL^−1^, and PFBA from 0.08 to 87 ng.mL^−1^. PFBS, PFOA, and PFBA concentrations in newborns decreased by almost half compared to maternal serum. Similar concentrations were also measured in placenta samples. Using high-resolution MS (HRMS), 49 novel PFASs classified into 11 classes were identified in the primary screening, of which 20 novel congeners in 4 classes were discovered in human blood and placentas for the first time. The concentrations of most novel PFASs were higher in placentas and cord serum compared to maternal serum. In addition, the presence of novel PFASs in cord serum may have affected neonatal birth outcomes and serum thyroid hormone, sex hormone, and glucocorticoid levels [117].

### 5.3. Breast Milk

According to the World Health Organization, infants should be exclusively breastfed for the first 6 months [118]. Mothers who cannot or do not want to breastfeed replace milk with formula, either in liquid form or diluted with drinking water. Infant formula manufacturers try to minimize the differences between infant formula and breast milk, and it is important to know that liquid sources of nutrition are safe for the baby. PFASs can be transmitted from mother to child through breastfeeding. Infants can also be exposed to PFASs through infant formula. A review article by LaKind et al. [119] compared concentrations of PFOA, PFOS, PFNA, and PFHxS in breast milk and infant formula with screening values for the listed PFASs in drinking water in children. Results from an earlier study confirmed by more recent data confirm that PFAS concentrations in breast milk are higher than screening levels for drinking water in children [120]. This is a global problem, and it is essential that pregnant women and breastfeeding mothers have the necessary data available to inform their future decisions about breastfeeding.

Lamichhane et al. [121] studied the effect of maternal PFAS exposure on breast milk lipid composition, as well as the combined effect of exposed breast milk and breast milk lipid composition on infant growth. PFAS and lipid concentrations in maternal serum were measured in 44 mother–infant pairs using UPLC-Q/TOF MS. Lipidomic analysis of breast milk collected 2–4 days after delivery and in 3-month-old infants was also performed. Fecal biomarkers calprotein and beta defensin 2 were measured in the stool of infants aged 3, 6, 9, and 12 months to assess intestinal immunomodulatory function in the infant intestine. High PFAS exposure caused an increase in the ratio of acylated saturated and polyunsaturated fatty acids in triacylglycerols. Altered phospholipid composition due to high PFAS exposure has been linked to slower growth in infants. Maternal exposure to PFASs affects the quality of breast milk, which may affect the health and growth of children [121].

A Canadian study observed that the use of personal care products (e.g., hairsprays and gels, nailcare products, fragrances and perfumes, makeup, hair dye) may be associated with higher plasma concentrations of PFOA, PFOS, and PFHxS. Similar results were observed in breast milk. The study used prenatal plasma from 1.940 women at 6–13 weeks of gestation and human milk from 664 women at 2–10 weeks postpartum. The frequency of use of personal care products was monitored during the first and third trimesters, 1 to 2 days postpartum, and 2 to 10 weeks postpartum. Increased use of nail care products, fragrances, makeup, hair dye, hair sprays, and hair gels was observed in the first trimester, which caused increased plasma concentrations of PFOA and PFOS. Similar results were obtained in the third trimester in plasma and in milk 2–10 weeks postpartum with the use of personal care products. In addition, the use of colored permanent hair dye 1–2 days postpartum may have caused higher concentrations of PFOA, PFOS, and PFNA in postpartum milk [122].

## 6. Health Effects

PFAS exposure is associated with adverse health risks such as cancer, steroid hormone disruption, infertility, lipid and insulin dysregulation, higher cholesterol levels, liver and kidney disease, altered immunological and thyroid function, and cardiovascular effects [36,123,124]. In infants and children, PFAS exposure can cause adverse effects on infants and premature babies and can lead to reduced growth parameters, lower visual motor skills and attention-deficit/hyperactivity disorder (ADHD) in childhood, lower levels of antibody concentrations against mumps and rubella, reduced lung and respiratory function, along with increased levels of glucocorticoids, progestogens, and uric acid [125,126,127,128].

**Figure 3:**
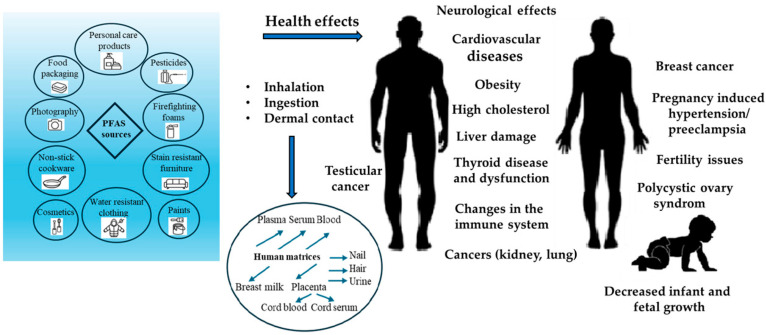
PFAS sources, routes of exposure, and health effects on humans.

The various sources, routes of exposure to PFASs, and their adverse health effects on human health are illustrated in Figure 3.

PFOS and PFOA are representatives of PFASs, which are toxic to humans and animals. Due to their multiple toxicity, they have been banned in many countries and replaced by substitutes, which, however, have shown even higher toxicity. The most pronounced toxicity has been reproductive toxicity. Epidemiological studies have shown that PFOS and PFOA can cause a decrease in testosterone levels in humans, abnormal levels of sex hormones, and an increase in the risk of infertility. PFASs can be absorbed through the intestinal or respiratory tract and subsequently cause reproductive disorders such as testicular cancer, testicular dysplasia syndrome, and menstrual disorders [129].

Wee et al. provide a detailed review of the sources, transport routes, and potential toxicological impacts of PFASs. In addition, the article provides an overview of the adverse effects of PFASs on human health [130].

The Centers for Disease Control (CDC, 2024) and the Agency for Toxic Substances and Disease Registry (ATSDR, 2021) reported that less consistent data are associated with effects on the immune system, disruption of thyroid hormones, and increased risk of cancer [12,131].

### 6.1. Lipid Metabolism

The Agency for Toxic Substances and Disease Registry [132] stated that the most consistent health effect of PFASs is increased cholesterol in the adult population. The relationship between lipid levels and PFAS exposure was also presented in the study by Liu et al. [85]. In the study, 575 human serum samples were collected. A total of 11 of 18 PFASs were detected in more than 80% of the samples. The highest serum concentrations were measured for PFOA (11.34 ng.mL^−1^) and PFOS (7.64 ng.mL^−1^). With increased exposure to PFASs, total TC and LDL concentrations also increased, while HDL and TG concentrations were not affected. PFUnDA and PFTrDA had a greater effect on blood lipid concentrations than other PFASs [85].

In a review article, Ho et al. [133] comprehensively assessed the effects of PFA exposure on LDL, HDL, total cholesterol (TC), and triglyceride (TG) concentrations in human plasma, serum, and whole blood. The study found a positive relationship between PFOA-LDL, PFOA-TC, PFOS-TC, and PFNA-LDL. The relationships between PFASs, especially perfluoroundecanoic acid (PFUnDA), and triglycerides tended to be negative [133].

Gardener et al. [134] investigated the relationship between serum concentrations of PFDA, PFNA, PFOS, PFOA PFHxS, and fasting serum concentrations of total cholesterol, triglycerides, and insulin. The pilot study (National Children’s Study) included 433 pregnant women whose serum was collected in the third trimester. PFASs were examined in quartiles in relation to serum biomarkers, gestational age at birth, and birth weight standardized for gestational age. Results showed that total cholesterol was positively associated with PFDA, PFNA, PFOS, and triglycerides, but PFASs were not associated with fasting insulin. PFNA was associated with an increased likelihood of preterm birth (<37 weeks of gestation) [134].

A large Japanese study by Hasegawa et al. [84] investigated the association between 28 PFASs and lipid concentrations in both maternal and cord blood. The analysis included 20.960 pregnant women. Data were obtained from a nationwide prospective general population birth cohort study (the Japan Environment and Children’s Study). Plasma samples were collected before 22 weeks of gestation to determine PFAS concentrations. Serum samples collected before, during, and after 22 weeks of gestation, at birth, and from cord blood were used to determine total cholesterol and triglycerides. Using linear regression models, 7 PFASs were quantified in more than 80% of the women out of the 28 PFASs analyzed, namely PFOA, PFNA, PFDA, PFOS, PFUnA, PFHxS, and PFTrDA. Of these, six had positive associations with maternal total cholesterol before 22 weeks of gestation but no association with cord blood TC. In the case of triglycerides, a negative relationship was shown between three PFASs and maternal blood and a positive relationship between four PFASs and TGs in cord blood [84].

PFAS exposure may affect lipid metabolism in pregnant women. Elevated plasma triglyceride levels may cause preeclampsia or hypertension. In an epidemiologic study by Yang et al., 11 PFASs were measured in the serum of 436 pregnant women and investigated the association with lipid parameters, namely TC, TG, HDL, LDL, PFOS, PFOA, PFHxS, PFUdA, PFNA, PFDA, and PFHpS were detected in serum with a detection rate of more than 70%. The following associations were found: positive association of PFHxS with TC, HDL, and LDL; and negative association of PFUdA with HDL, PFDA with LDL, and PFOA, PFNA, PFDA, and PFUdA with LDL/HDL. The results indicate the possibility of an impact of PFAS exposure on lipid metabolism in pregnant women [135].

In an Italian study, there was a cross-sectional analysis conducted of 319 pregnant women aged 14–48 years who came from areas with high exposure to PFASs through drinking water. Concentrations of PFOA, PFOS, and PFHxS, total cholesterol (TC), HDL-C, and LDL-C were determined. Elevated TC, LDL-C, and HDL-C values increased gradually and may have adverse effects on both the mother and the fetus. Associations between PFASs and lipid markers varied throughout all trimesters of pregnancy. In the first trimester, the relationship between PFOS and TC and between PFHxS and HDL-C was positive and similar to that in non-pregnant women, with a reversal during the third trimester. There was an inverse relationship between PFOA and PFHxS and TC and LDL-C. The results highlight the importance of the correct timing of PFAS measurements during pregnancy [136].

### 6.2. Diabetes Mellitus

Exposure to persistent organic pollutants in the environment may be one of the risk factors for the development of diabetes mellitus, which has a growing prevalence worldwide.

In the case–control study [137], there was investigated the relationship between PFAS exposure and the risk of developing type 2 diabetes mellitus (T2DM). A total of 252 T2DM cases and 252 controls were examined, and the results showed that serum PFHxS and PFHpA were significantly positively associated with the risk of developing T2DM at the lower levels [137].

Xu et al. [138] tested the hypothesis that PFASs can cause gestational diabetes mellitus (GDM) by modulating glucose metabolism. The case–control study included 171 women with GDM and 169 controls and determined 15 PFASs, including homologs of PFOA and PFOS, precursors of PFSA and PFCA, and alternatives of PFOS and PFOA, which were detected in more than 70% of maternal serum samples. The highest concentrations in maternal serum were measured for PFOA 7.43 ng.mL^−1^, PFOS 4.23 ng.mL^−1^, and chlorinated polyfluorinated ether sulfonate in the ratio 6:2 (6:2 Cl-PFESA). Exposure to PFOA, PFNA, PFDA, PFUnDA, PFDoA, PFHxS, and PFOS was significantly higher in women with GDM compared to controls. Concentrations of PFOA, PFOS, PFUnDA, PFDoA, and 6:2 Cl-PFESA were associated with impaired glucose homeostasis in pregnancy and an increased risk of GDM, while an inverse association with GDM was found for PFHxS, 4:2 FTS, and 6:2 FTS [139].

A Korean epidemiologic study [139] presented the relationship between serum PFAS concentrations and the prevalence of prediabetes and prediagnostic diabetes. This is the first study to describe a strong positive association between serum PFAS concentrations and prediabetes and the prediagnostic stage of diabetes. The study included 2709 participants aged ≥ 19 years. Prediagnostic diabetes and prediabetes were determined based on glycated hemoglobin HbA1c values. Serum PFOA, PFNA, PFDeA, PFHxS, and PFOS were determined by HPLC combined with tandem mass spectrometry. Concentrations of individual PFAS compounds, as well as mixtures, were associated with a higher risk of prediabetes. Significant positive associations were found between serum PFOS and PFHxS and increased HbA1c values and a higher risk of prediagnostic diabetes. The results suggest that PFAS exposure may contribute to impaired glucose homeostasis in the prediabetic stage and thus to the development of diabetes [139].

A Chinese case–control study [44] included 495 women, 165 with GDM and 330 controls. GDM was measured between 24 and 28 weeks of gestation by glucose test. PFOA, PFOS, PFBS, PFDoA, PFUA, PFDA, PFHpS, PFNA, PFHxS, PFDS, PFHpA, and PFOSA in serum were determined by UPLC-Q/TOF MS. PFHpA, PFDS and PFOSA were all present in ≤ 80% of samples and were therefore not considered further. PFOS, PFOA, PFBS, and PFDoA were measured at significantly higher concentrations in maternal serum in early pregnancy, which may be associated with a higher risk of GDM [44].

### 6.3. Disorders in Women

There are many studies on PFAS exposure and male fertility, and most of them show that PFAS exposure leads to impaired male fertility [140]. The available results obtained from studies focusing on women have been quite inconsistent. Therefore, more systematic reviews and meta-analyses have been conducted to examine the evidence on the effect of PFAS exposure during pregnancy on maternal fertility, in addition to quantitative assessment of maternal PFAS concentrations during pregnancy and the risk of fertility and infertility.

Therefore, Wang et al. [141] conducted a meta-analysis in a review article. A total of 5468 records from four databases were searched, and after gradual elimination, 13 articles that fully met the inclusion criteria were left and were used for the meta-analysis. The results showed that exposure to PFOA was negatively associated with female fertility and positively associated with the odds ratio for infertility. Exposure to (PFOS) was negatively associated with the odds ratio for fertilization. The obtained effect values for exposure to PFNA, PFDA, and perfluorohexanesulfonate (PFHxS) did not provide sufficient evidence of an association with female fertility [141].

Preeclampsia is a condition of hypertensive disorder during pregnancy that can be a cause of maternal mortality or can be associated with low birth weight, prenatal death, and preterm birth. Tian et al. investigated in a case–control study [142] the possible association between preeclampsia and serum PFAS concentrations in 82 women with preeclampsia and 169 healthy women. A total of 15 PFASs were analyzed in maternal serum before delivery. Concentrations of PFOA and 6:2 Cl-PFESA were associated with an increased risk of developing preeclampsia, especially in primiparous women expecting a girl. Increased PFOA concentrations were significantly associated with increased blood pressure. In relation to neonatal development, a negative relationship was found between exposure to PFOS, PFNA, PFUnDA, and 6:2 Cl-PFESA and birth weight. Regression models indicated that significantly higher risks of low birth weight occurred in women with preeclampsia compared to normal pregnancies. This observational study considered not only legacy PFASs but also new alternatives and included a low birth weight parameter to better elucidate the possible effects of PFAS exposure on adverse birth outcomes [142].

Preeclampsia was also addressed in a study by Thompsom et al. [143], which examined the association between hypertensive disorders of pregnancy (preeclampsia and gestational diabetes) and exposure to four PFASs: PFOA, PFOS, PFHxS, and PFNA in a sample of 513 African American mothers. Serum samples were collected from mothers between 8 and 14 weeks of pregnancy (from the Atlanta African American Maternal–Child cohort). Regression models were used to assess associations. Mean PFOS and PFHxS levels were lower in patients with preeclampsia compared to patients without hypertensive disorders. PFAS concentrations were not associated with gestational hypertension or preeclampsia. In this study, no association was found between serum PFAS concentrations measured in early pregnancy and hypertensive disorders of pregnancy [143].

There is an association between PFASs and reduced birth weight, but it may be complicated by glucose status due to the effects of PFASs on fetal growth and placental transport. Wang analyzed data from 1405 mother–child pairs from prospective time-to-pregnancy (TTP) cohort studies and investigated whether maternal glucose levels were associated with prenatal PFAS exposure and birth weight. Plasma concentrations of PFOA, PFOS, PFNA, PFDA, PFUA, and PFHxS were determined in the first trimester. Plasma glucose was measured at 24–28 weeks of gestation. The results suggest that infants born to mothers with hyperglycemia may be sensitive to PFAS exposure [115].

Zhang et al. investigated whether PFAS exposure is associated with polycystic ovary syndrome (PCOS). The prospective cohort study included a total of 502 women from the Environment and Reproductive Health Study who were undergoing reproductive treatment at a fertility clinic. Nine PFASs were measured in serum samples. Higher serum concentrations of PFOS and PFHxS were associated with an increased risk of PCOS. No associations were found for PFOA [144].

The results of this study were consistent with the results of a hospital-based case–control study by Zhan et al., which included 366 women with PCOS and 577 controls. Interestingly, the concentrations of PFOA were several times higher than in the discussed article (7.2 versus 1.7 ng.mL^−1^), while the concentrations of PFOS (3.9 and 3.5 ng.mL^−1^) and PFHxS (0.22 and 0.8 ng.mL^−1^) were found. The results support the possible adverse effects of PFASs on female reproductive function, but further larger studies are needed to confirm the findings [145].

The review article by Yi et al. summarizes the impact of PFAS exposure on the development of four reproductive health outcomes in women studied to date, namely polycystic ovary syndrome, endometriosis, primary ovarian insufficiency, and diminished ovarian reserve [146].

### 6.4. Thyroid Hormones

Thyroid hormones play an important role in regulating health, brain development, depression, and obesity. Thyroid hormone deficiency during pregnancy can cause reduced IQ or neurological damage in children. In addition, it can cause intellectual disability or growth retardation in adolescents. PFASs are potential thyroid disruptors [147,148].

He et al. [149] investigated the relationship between exposure to 30 PFASs and thyroid function in 194 Chinese children aged 3–17 years using multiple statistical models. Free triiodothyronine, free thyroxine (FT4), and thyrotropin were tested as indicators of thyroid function, and subclinical hypothyroidism was diagnosed. PFASs and their alternatives were associated with changes in thyroid hormone levels and subclinical hypothyroidism. Higher concentrations of perfluorohexanoic acid (PFHpA) caused a decrease in FT4 levels and an increased likelihood of subclinical hypothyroidism. The measured PFOA concentration (median) of 23.22 μg.L^−1^ was comparable to some previous studies. For example, in the first American large-scale report, the concentration of 29.3 μg.L^−1^ was found in children aged 1–17 years [150], and in adults, 23.1 μg.L^−1^ [151]. In a prospective Korean cohort study, the concentration of PFOA in pregnant women was 23.22 μg.L^−1^ [152].

Linear regression models were used to evaluate data from the China National Human Biomonitoring in a cross-sectional study investigating the associations between PFAS exposure and thyroid hormone levels. Eight PFASs were monitored in 10,853 serum samples. The median concentrations of PFOS (4.31 μg/L) and PFNA (1.79 μg/L) in adults were similar to those in other studies, with PFOS and PFNA concentrations of 5.17 μg/L and 1.00 μg/L in adults [153], and 7.78 μg/L and 1.01 μg/L in adolescents [154]. Higher serum PFOA concentrations were associated with decreased FT4 levels and increased TSH levels. A birth cohort study in Spain found opposite results in adolescent boys aged 15–17 years [155].

In a U.S. study by Lewis [156], higher serum PFOA levels were associated with lower TSH levels in adolescent females, which are different results from the discussed study in children. The data were obtained from the National Health and Nutrition Examination Survey. These different results may be related to variations in the regions and populations of each study.

In the prospective cohort study by Lebeaux et al., the authors investigated the association between maternal serum concentrations of PFASs (PFOA, PFOS, PFNA, and PFHxS) during pregnancy with levels of thyroid-stimulating hormone (TSH), total thyroxine (TT4), total triiodothyronine (TT3), free thyroxine (FT4), and free triiodothyronine (FT3) measured in maternal and cord serum. The study involved 468 pregnant women and their children. The results show that there is no strong association between maternal serum PFAS concentrations measured in the second trimester and maternal and cord serum thyroid hormones [150]. A cross-sectional study by Xie et al. examined thyroid function parameters and serum PFAS concentrations in adolescents. Concentrations of 18 PFASs were measured, including 11 PFCAs, four PFSAs, and three novel PFASs (4:2, 6:2, and 8:2 Cl-PFESA). The epidemiological study included 836 adolescents aged 11–15 years who came from a high-PFCA-exposed area near a fluorochemical industrial plant. Elevated FT3 and decreased FT4 levels were found. Using logistic and linear regression, a significant negative correlation was found between PFOA and FT4 and a positive correlation between PFHxS and FT3, with both PFOA and PFHxS being assessed as risk factors [157].

Wang et al. conducted a case–control study to investigate the effect of PFAS exposure on serum metabolome and its association with thyroid cancer. The study included 746 thyroid cancer cases and 746 controls. LC-MS/MS was used to determine the concentrations of PFDoA, PFUnDA, PFDA, PFNA, PFOA, PFOS, PFHxS, PFHxA, PFHpA, PFBS, and PFPeA. Exposure to PFHxA and PFDoA was associated with an increased risk of thyroid cancer, while PFOA and PFHxS were associated with a decreased risk [158].

### 6.5. Liver Disorders

#### 6.5.1. General Liver Toxicity

PFASs are known to accumulate in various organs. Many studies have shown an association between PFAS exposure and liver toxicity, as well as the impact of PFASs on liver homeostasis, lipid and bile acid metabolism, and hepatocarcinogenesis. In a review article, Maerten et al. discuss the role of PFASs in liver toxicity and the mechanisms underlying the hepatotoxic effects of PFASs and fill the knowledge gap for a better assessment of PFAS risks [159].

In humans, the highest concentrations of PFASs were measured in the liver, kidneys, and lungs. PFOS predominates in the liver, while PFBA predominates in the lungs and kidneys. Many studies [160,161,162,163] reported that exposure to PFASs (PFOS, PFOA, PFNA, PFHxS, fluorohexanoic acid, perfluoro-3,5,7,9,11-pentaoxadocanoic acid, and 6:2 chlorinated polyfluorinated ether sulfonate (6:2 Cl-PFESA)) can lead to liver damage in humans. Markers of liver damage include alanine aminotransferase (ALT), aspartate aminotransferase (AST), gamma-glutamyltransferase (GGT), alkaline phosphatase, and serum bilirubin, the increased values of which cause an increase in inflammatory markers. PFOS, PFOA, and PFBA increase ALT activity [164].

#### 6.5.2. Dyslipidemia, Steatosis, and Steatohepatitis

These articles summarize studies that have evaluated the relationship between serum PFASs and steatosis and steatohepatitis as a consequence of dyslipidemia. PFNA correlates with LDL, PFOS with total cholesterol, and PFOA with both LDL and TC [133,165,166].

Yang et al., in a cross-sectional study, described the potential role of PFASs in the comorbidity of hepatic steatosis among patients with acute coronary syndrome. This correlation study provides a reasonable opportunity to hypothesize the mechanisms behind the comorbidity of hepatic steatosis and inform tertiary prevention strategies [166].

A systematic review of 58 epidemiological studies presented evidence of the association between PFAS exposure and higher concentrations of LDL, HDL, and total cholesterol (TC), particularly for PFOA-LDL, PFOA-TC, PFOS-TC, and PFNA-LDL. Moreover, a negative association was observed between PFUnDA and triglycerides (TGs) [133].

In a long-term study involving 1420 older participants (≥60 years), it was observed that PFOA and PFNA might be associated with nonalcoholic fatty liver disease [167].

PFOS, PFOA, and PFHxS have been associated with an increased risk of developing steatohepatitis [159,168]. PFAS exposure can lead to hepatic steatosis, which is manifested by increased inflammatory markers and biomarkers of liver injury.

#### 6.5.3. Hepatocarcinogenesis

The International Agency for Research on Cancer considers both PFOS and PFOA to be probable human carcinogens in humans at high exposure and in humans with testicular and kidney tumors. The association of PFOS and PFOA with hepatocarcinogenesis has been demonstrated in laboratory animals but not in humans. In conclusion, it can be stated that exposure to PFASs can cause an increase in serum markers of liver damage, an increase in cholesterol and hepatic lipid accumulation in humans, and interference with bile acid metabolism by affecting hepatic transporters [132].

The epidemiological study by Dai et al. investigated the correlations between PFAS concentrations and biomarkers of liver function. The study included 227 patients, of whom 197 had hepatocellular carcinoma and 30 suffered from severe liver disorders such as cirrhosis and hepatitis. A total of 21 PFASs were determined, with the highest concentrations in serum, then in blood and urine. The concentrations were higher in the group of patients with HCC. Higher concentrations of PFASs are achieved in men compared to women. The highest concentrations in blood and serum were PFOA, PFOS, PFBS, and PFHxS. The highest concentration was determined in urine for PFBA and PFPeA. The concentrations of PFBS, PFHpS, and PFHxPA correlated with increased values of alkaline phosphatase (ALP), aspartate aminotransferase (AST), and alpha-fetoprotein (AFP) (*p* < 0.05). The results provide a good basis for further study on the possible hepatotoxicity of PFASs [169].

### 6.6. Hypertension

PFAS exposure is associated with adverse health effects, such as dyslipidemia, hypertension, and hypertensive disorders of pregnancy (HDP), specifically preeclampsia and gestational hypertension. The link between PFASs and HDP is discussed in a review article by Erinc et al. [170].

Gestational hypertension (GH) is new-onset hypertension occurring after 20 weeks of pregnancy. A Chinese birth cohort study evaluated the relationship between 13 PFASs and gestational hypertension and blood pressure (BP) during pregnancy. A total of 826 pregnant women were included, with blood samples collected at 16 weeks of gestation. The highest concentrations of 11.99, 8.81, and 5.43 ng.mL^−1^ were measured for PFOA, PFOS, and PFHxS, respectively. A total of 5.57% of women developed GH. A lower probability of GH was demonstrated for PFOS, PFDA, PFUdA, and PFDoA. Associations were found between PFASs and lower systolic and diastolic blood pressure values in the third trimester. PFDA and PFUdA affected systolic blood pressure only in pregnant women whose fetuses were female [171].

In the Project Viva longitudinal pre-birth cohort study [172, there was investigated the relationship between PFAS exposure and hypertensive disorders of pregnancy (i.e., preeclampsia, gestational hypertension), which can cause maternal and neonatal morbidity and mortality. The study included 1558 pregnant women and measured PFHxS, PFOS, PFOA, PFNA, PFDA, EtFOSAA, MeFOSAA, and PFOSA in plasma samples. The parameters examined were preeclampsia, gestational hypertension, and trimester-dependent diastolic and systolic blood pressure. An association was found between PFOS, PFOA, and PFHxS concentrations and gestational hypertension, but not with preeclampsia. Analysis of the PFAS mixture indicated a positive association with gestational hypertension, while the associations for PFOA and PFHxS remained. Furthermore, a positive association was observed between high concentrations of PFOS and PFOA and diastolic blood pressure in the second and third trimesters, as well as for the PFAS mixture in both trimesters. For systolic blood pressure, an association was demonstrated only for PFOA in the second and third trimesters. The results indicate a possible impact of PFAS exposure on blood pressure regulation during pregnancy [172].

### 6.7. Cancer

The International Agency for Research on Cancer classified PFOA as a Group 1 carcinogen and PFOS as a Group 2B carcinogen in 2023 [173]. The United States Environmental Protection Agency (EPA) has set a recommended health level of 70 ng.L^−1^ for lifetime exposure to PFOS and PFOA [174]. Given the increasing number of epidemiological studies on PFASs and their adverse effects on humans, it has become imperative to address the carcinogenic mechanisms. The aim of the review by Zheng et al. was to update the evidence on the association between PFAS exposure and cancer. It presents a review of the literature published between 2019 and 2024 on the potential relationship between PFASs (PFOS, PFOA, PFHxS, PFHxA, and PFNA) and various types of cancer, such as breast, testicular, prostate, colorectal, kidney, liver, lung, thyroid, leukemia, melanoma. In addition, the article also addresses potential carcinogenic mechanisms such as endocrine disruption, lipid metabolism, epigenetic alteration, oxidative stress, immunosuppression, and chronic inflammation [158,159,175,176,177,178,179,180,181,182].

### 6.8. Obesity

Based on the data from the National Health and Nutrition Examination Survey, Chen et al. investigated the effects of PFAS exposure (PFOA, PFOS, PFUdA, PFNA, PFHxS, PFDeA, and 2-(N-methyl-perfluorooctane sulfonamide) acetic acid) on obesity. A total of 11,090 individuals were analyzed, and linear and logistic regression models were used to evaluate the effects. The role of inflammatory markers (neutrophils, lymphocytes, and alkaline phosphatase) and oxidative stress markers (gamma-glutamyltransferase, total bilirubin, and uric acid) was also examined. Lymphocytes, alkaline phosphatase, and total bilirubin were significantly associated with both obesity and type 2 diabetes mellitus. PFAS exposure was positively associated with the development of obesity and T2DM and mediated by inflammation and oxidative stress. PFNA was positively associated with obesity and T2DM, while PFOA was negatively associated with T2DM. PFDeA was positively associated with hypertension but negatively with obesity and T2DM [183].

Averina et al. investigated associations between PFASs and dyslipidemia, hypertension, and obesity in an adolescent population-based cohort. A cross-sectional study was conducted on 940 adolescents with a mean age of 16.4 years who had either hypertension, obesity, or dyslipidemia. Serum concentrations of PFHxS, PFOA, PFOS, PFNA, PFDA, and PFUnDA were measured by UHPLC-MS/MS. The results showed a positive association of PFOS, PFNA, PFDA, and PFUnDA with apolipoprotein B, LDL, and total cholesterol. Concentrations of PFOS and PFOA were positively associated with the risk of hypertension, and PFHxS and PFHpS with obesity [184].

In a cross-sectional study by Geiger et al., the association between PFOA and PFOS and BMI and waist circumference was investigated in a representative sample of 2473 American children. Elevated concentrations of PFOA may indicate an association with overweight and obesity in children. This topic requires further research, which will include, for example, abdominal adiposity, fat distribution, etc. [185].

### 6.9. Autism

Autism spectrum disorder (ASD) is a complex of neurodevelopmental disorders characterized by deficits in social interaction and communication, as well as the presence of restricted, stereotyped interests and repetitive behaviors [186]. Although there is a possibility of a potential association between PFASs and the development of ASD, large studies are needed to confirm this. Six case–control studies and one cohort study have evaluated the possible association between PFASs and the development of ASD. The results have been highly inconsistent.

The population-based nested case–control studies by Liew et al. (220 with ASD and 550 controls) and Lyall et al. examined prenatal exposure to PFASs and the association with ASD (533 children with ASD and 433 controls) and found no consistent evidence that maternal plasma concentrations of PFASs were associated with an increased risk of ASD in a Danish population [187,188]. Long et al. measured PFAS levels in the amniotic fluid of 75 children with ASD and 135 controls and found an inverse association between PFASs and risk of ASD, which may be related to the weak estrogenic activities of PFASs [189]. Skogheim et al. studied 400 children with ASD and 980 controls and confirmed an association between PFOA and ASD in boys [190]. The obtained data within the study were based on the Norwegian Mother, Father, and Child Cohort Study.

In the high-risk autism spectrum disorder cohort, Oh et al. investigated the association between prenatal exposure to PFASs and an increased risk of autism spectrum disorders. The study included 173 mother–child pairs, with children aged 3 years confirmed to have autism spectrum disorders. Nine PFASs were measured in maternal serum, namely PFOA, PFOS, PFHxS, PFNA, PFDA, PFUnDA, PFDoDA, MeFOSAA, and EtFOSAA. Positive associations were found for PFOA and PFNA and an increased risk of autism spectrum disorders, while PFHxS showed negative associations. Given that exposure to individual and combined PFASs had different effects on the risk of ASD depending on the advanced age of the mother at delivery, further study with a larger sample size is needed [191].

### 6.10. Respiratory Diseases

The term lung function includes respiratory, metabolic, defense, and other functions of the lungs. Changes in lung function indicate the risk and severity of clinical symptoms and their impact on quality of life. Children, as a risk population, are more likely to be exposed to PFASs due to higher respiratory rates, interactions with contaminated surfaces (e.g., crawling on the floor), etc. As a result of environmental exposure to PFASs in children, respiratory burden arises, which raises concerns about the rapid development of pulmonary systems [192].

It is very important to determine the relationship between PFAS exposure and respiratory diseases, such as asthma. Meta-analysis processed bibliographic data from various epidemiological studies that were dedicated to diagnosing asthma in children under 17 years of age, revealing a connection between PFOA exposure and a higher risk of developing asthma as well as between PFOS and lung function disorders [193].

Exposure to PFASs in pregnant women can cause respiratory problems in children after birth. Among the main factors that affect lung development in the prenatal period are, for example, premature birth, placental insufficiency, maternal tobacco smoking, etc.

Kung et al. [192], in their cohort study, investigated the relationship between intrauterine and postnatal PFAS exposure and lung function in children. The study used 165 umbilical cord blood plasma and serum samples from 8-year-old children. The average concentrations of PFOA, PFOS, PFNA, and PFUA were determined by HPLC-MS/MS. The monitored lung functions were FEV1 (forced expiratory volume in one second), FVC (forced vital capacity), PEF (peak expiratory flow), and FEV1/FVC. The average concentrations of selected analytes were higher in umbilical cord blood than in serum. PFASs were not shown to significantly affect lung function in children. However, they found that PFOS concentrations were significantly inversely correlated with lung function in children with lower birth weight and suffering from allergic rhinitis [194].

### 6.11. Kidney Diseases

Chronic kidney disease has a high incidence and mortality rate, especially in patients with diabetes and hypertension. PFASs may be hazardous to kidney function. Liang 2023 et al. [195] investigated the relationship between PFOAS, PFOS, and Cl-PFESA, heavy metals (cadmium, lead, arsenic) to glomerular filtration rate and chronic kidney disease. PFOA and heavy metals were positively correlated with chronic kidney disease. PFOA, PFOS, CI-PFESA, and arsenic were negatively associated with glomerular filtration rate.

The epidemiological study provided epidemiological evidence that Cl-PFESA alone and together with heavy metals may contribute to kidney damage [196].

A nationwide cross-sectional study was conducted on 13,979 adults in the U.S. to examine the association of serum PFASs with the risk of uric acid and hyperuricemia. Even at low concentrations, the dominant PFOA may have caused an increase in uric acid and an increased risk of hyperuricemia and contributed to a decline in kidney function [194].

### 6.12. Cardiovascular Diseases

PFASs can play a role in the development of cardiovascular diseases (myocardial infarction, ischemic stroke, heart failure). A Swedish epidemiological study examined the association between PFAS exposure and cardiovascular disease in 2278 participants aged 45–75 years. PFOA, PFOS, and PFHxS were measured in plasma. A second independent study measured PFHxS, PFOA, linear isomer of PFOS, PFNA, PFDA, and PFUnDA in plasma in 1016 participants aged 70 years. Neither study found an increased risk of cardiovascular disease at slightly elevated levels of PFASs [197].

Potential associations between PFASs and values of pre-selected proteomic biomarkers in plasma were observed in a Swedish epidemiological study by Dunder et al. PFOA, PFOS, and PFHxS were measured by untargeted metabolomics, and 249 proteomic biomarkers in plasma in 2342 participants were measured by proximity extension assay. Epidermal growth factor receptor (EGFR) and paraoxonase type 3 (PON3) levels were positively associated with all three PFASs, while resistin (RETN) and urokinase surface plasminogen activator receptor (uPAR) showed inverse associations with all three PFASs [198].

### 6.13. Bone Metabolism

PFAS exposure is associated with a number of disorders and may also act as a toxic agent for the skeleton. PFASs interfere with many hormones that affect bone metabolism. An association has been found between PFAS exposure and lower bone mineral content and bone mineral density in children and adolescents [197]. A birth cohort study in Ohio included 197 adolescents aged 12 years, from whom serum was collected. Concentrations of PFOA, PFOS, and PFNA were determined using the SPE-LC-MS/MS method. Higher concentrations of PFASs caused a decrease in the BMC (bone mineral content) z-score. The adverse effects of PFASs can be mitigated by higher calcium intake, increased physical activity, and a better-quality diet [199].

### 6.14. Neurodevelopment in Child

PFASs can cross the placenta, and prenatal exposure to PFASs has been associated with neurodevelopmental disorders in children, increased risk of neuropsychological problems, and abnormal behavior in adulthood. The effects of PFASs on infant neurodevelopment were investigated in an epidemiological study by Zhou et al., which included 1285 mother–infant pairs. PFOS, PFOA, PFHxS, and 62Cl-PFESA were confirmed in more than 90% of samples. Each increase in PFAS concentration resulted in poorer communication domain scores. The decrease in communication domain scores in 6-month-old infants was mainly attributed to the increase in PFOS concentration. The findings indicated that PFASs may adversely affect neurodevelopment in children [200].

An association between prenatal exposure to legacy PFASs and children’s intelligence and executive functioning was observed in a Canadian prospective multi-site Maternal Infant Research on Environmental Chemicals (MIREC) cohort study. An association with child gender was also examined. Plasma concentrations of PFOA, PFOS, and PFHxS were measured in the first trimester, and 522 children were assessed for full-scale performance and verbal IQ using the Wechsler Preschool and Primary Intelligence Scale. Results indicated that each two-fold increase in prenatal exposure to PFOA, PFOS, and PFHxS was inversely associated with performance IQ, but only in males. No significant association was found in females [201].

### 6.15. Cerebral Palsy

It is known that PFASs can be transported to the fetus across the placenta. Thyroid hormone levels may be disrupted during pregnancy. Insufficient hormone levels during a critical stage of brain development can lead to neurodevelopmental disorders such as cerebral palsy (CP), which is one of the most common physical and motor disabilities in childhood. The author Vilhelmsson et al. [202], in a case–control study, investigated the association between prenatal PFAS exposure and the risk of CP. The study included 322 CP cases, 258 premature infants, and 343 controls. Serum samples collected at 10–14 weeks of gestation were obtained from a biobank, and concentrations of PFOA, PFOS, PFNA, and PFHxS were determined. No associations were observed between prenatal exposure to PFASs and the risk of CP, except in premature infants, but the results were not entirely consistent and therefore require further study.

The Swedish study discussed was preceded by only one study that investigated whether prenatal exposure to PFASs increases the risk of congenital cerebral palsy. This Danish epidemiological study followed 83,389 newborns and mothers, identified 156 CP cases, and randomly selected 550 controls. A total of 6 PFASs were identified in more than 90% of samples, and 16 PFASs in maternal plasma were collected in early and mid-pregnancy. Higher concentrations of mainly PFOS and PFOA in maternal plasma increased the risk of CP in boys. In girls, no association was found between PFASs and CP [203].

## 7. Conclusions

PFASs are characterized by high toxicity, stability, and slow degradation, which causes long persistence in various components of the environment, from where they accumulate in the human body through plants and animals and cause adverse health effects. The toxicological properties of PFASs in humans have not been sufficiently studied due to the large number of compounds. Our review is based on scientific studies to clarify the presence of per- and polyfluoroalkyl substances in human matrices, such as hair, nails, urine, placenta, and breast milk, but especially to health problems caused by PFAS exposure. Exposure to these toxicants has caused a variety of adverse health consequences; therefore, various biomonitoring approaches in humans are necessary. Exposure data, together with modifiable environmental risk factors, are crucial for risk assessment and facilitate the development of new strategies to prevent and mitigate the adverse effects of PFASs.

The long-term persistence and bioaccumulation of PFASs require minimizing their use to protect both the environment and human health. A key step is to implement appropriate substitutes in various applications. In the textile industry, the use of silanes or hydrocarbon-based repellents should be considered instead of fluorochemicals. The non-stick cookware market could switch to alternatives such as ceramic, cast iron, stainless steel, or even diamond carbon coatings to eliminate PFASs. Additionally, PFAS-free packaging should be introduced for food packaging, and PFAS-free additives should be used in personal care products and other household items. Existing contamination requires immediate attention, and various remediation strategies are being explored to remove PFASs from water, soil, and other environmental media.

Another crucial step is to support innovation in manufacturing processes. Closed-loop recycling systems for PFAS-containing materials can minimize waste generation and environmental contamination. Collaboration between industry, researchers, and policymakers is essential in transitioning to PFAS-free alternatives. The industry can invest in exploring new alternatives, while research can contribute new materials. Public awareness campaigns are also essential to increasing consumer demand for PFAS-free products.

Such a combined approach, focusing on alternatives and remediation strategies, offers the best solution to the problem.

Exposure data, along with modifiable environmental risk factors, are key to risk assessment and will help develop new strategies to prevent and mitigate the adverse effects of PFASs.

## Figures and Tables

**Table 1:** Determination of PFASs in different biological matrices using LC/MS techniques.

Sample	Analyte	Pretreatment	Analytical Method	Ref.
Maternal serum	PFOS, PFOA	Extraction with ACN Sonication, centrifugation Next extraction with ACN	LC-MS/MS ESI-	[75]
Blood	PFOS, PFOA	Extraction with ACN Sonication, centrifugation Next extraction with ACN Purification on ENVI-Carb cartridge	UPLC-MS/MS ESI-	[76]
Human plasma	42 PFASs	Captiva EMR-Lipid (1 mL, 96-well plate, 40 mg sorbent mass)	LC-q-Orbitrap ESI-	[77]
Cord serum	19 PFASs	ACN Off-line SPE (Oasis WAX)	UHPLC-MS/MS ESI-	[78]
Plasma	PFOA, PFOS PFHxS, PFNA, PFDA, EtFOSAA, MeFOSAA	On-line SPE (Polaris C18 HD 10 mm × 2 mm)	HPLC-MS/MS	[79]
Cerebrospinal fluid	26 PFASs	LLE with ethyl acetate	UPLC-MS/MS ESI-	[80]
Breast milk	30 PFASs	Off-line SPE (Oasis WAX 6 mL/150 mg)	UPLC-MS/MS ESI-	[81]
Maternal, cord serum, breast milk	PFHpA, PFOA, PFHxS, PFOS, PFNA, and PFDA	On-line SPE (HyperSep C8-SE (7 μm))	HPLC-MS/MS ESI-	[82]
Serum	PFOA, PFNA, PFCA	Sonication with 0.5 M TBAS and NaHCO_3_/Na_2_CO_3_ (pH = 10) Shaking with MTBE	UPLC-MS/MS ESI-	[83]
Maternal, cord blood	PFDA, PFDS PFHxS, PFNA, PFOA, PFOS, PFTrDA, PFUnA	On-line SPE (Oasis WAX 2.1 × 20 mm, 30 μm)	LC-MS/MS ESI-	[84]
Human serum	18 PFASs	QuEChERS	UHPLC-Orbitrap MS ESI-	[85]
Whole blood	75 PFASs	Filter-assisted precipitation, 1 mL SPE filter cartridge with two filter frits; MeOH, ACN, IPA	LC-MS/MS ESI-	[86]
Plasma	PFHxS, PFOA, PFNA, PFOS, PFDA	On-line SPE (HySphere C8-SE, 7 µM)	HPLC-MS/MS ESI-	[87]
Plasma	14 PFASs	Off-line SPE (Oasis^®^Wax, 150 mg, 6 mL, 30 μm)	HPLC-MS/MS ESI-	[88]
Plasma, human milk	PFOA, PFOS, PFNA, PFDA, PFHxS PFHpA, PFHpS, PFHxA	On-line SPE (HySphere C8-SE, 7 μM) On-line SPE (Oasis WAX 30 µm, 10 mm × 1 mm)	HPLC-MS/MS	[89]
Plasma	17 PFASs	0.5 mol.L^−1^ tetrabutylammonium hydrogen sulfate + 0.25 mol.L^−1^ NaHCO_3_/Na_2_CO_3_ + MTBE	LC-MS/MS ESI-	[90]
Cord blood	24 PFASs	TBA + Na_2_CO_3_ + MTBE	LC-MS/MS ESI-	[91]
Plasma	56 PFASs	ACN Off-line SPE (Anavo^®^ HMR–Lipid SPE)	UHPLC-Q/Orbitrap HRMS	[92]
Serum	32 PFASs	On-line SPE (Strata-X- AW 20 × 2.0 mm, 25 μm)	UHPLC-Orbitrap HRMS	[93]
Serum, breast milk	PFOS, PFOA, PFNA, PFHpA, PFHxS, PFDA, PFUnDA, PFDoDA	On-line SPE (Strata RP, 2.1 × 20 mm)	UPLC-MS/MS ESI-	[94]

PFOA—perfluorooctanoic acid; PFOS—perfluorooctane sulfonate; PFCAs—perfluoroalkyl carboxylates; POSAs—perfluoroalkyl sulfonamides; PFNA—perfluorononanoic acid; PFDA—perfluorodecanoic acid; PFDS—perfluorodecane sulphonic acid; PFHxS—perfluorohexane sulphonic acid; PFTrDA—perfluorotridecanoic acid; PFUnA—perfluoroun-decanoic acid; PFHpA—perfluoroheptanoic acid; PFDoDA—perfluorodo-decanoic acid; PFHpS—perfluoroheptane sulfonic acid; PFHxA—per-fluorohexanoic acid; PFUnDA—perfluorooctane acid; PFSA—perfluoro-alkane sulfonic acid; PFCA—perfluoroalkyl carboxylic acid; MTBE—methyl tert-butyl ether; TBA—thiobarbituric acid; EtFOSAA—2-(N-ethyl-perfluorooctane sulfonamido) acetate; MeFOSAA—2-(N-methyl-perfluorooctane sulfonamide) acetate; IPA—hypergrade 2-propanol.

## Data Availability

Not applicable.
